# Optic Neuropathy Secondary to Polyarteritis Nodosa, Case Report, and Diagnostic Challenges

**DOI:** 10.3389/fneur.2017.00490

**Published:** 2017-09-20

**Authors:** Kristian A. Vazquez-Romo, Adrian Rodriguez-Hernandez, Jose A. Paczka, Moises A. Nuño-Suarez, Alberto D. Rocha-Muñoz, Maria G. Zavala-Cerna

**Affiliations:** ^1^Ophthalmology Department, Hospital Regional “Dr. Valentín Gómez Farías”, Zapopan, Jalisco, México; ^2^UIEC, Hospital de Especialidades, Centro Médico Nacional de Occidente (CMNO), Instituto Mexicano del Seguro Social (IMSS), Guadalajara, Jalisco, México; ^3^Unidad de Diagnóstico Temprano del Glaucoma, Guadalajara, Jalisco, México; ^4^Centro Universitario de Tonala (CUTONALA), Universidad de Guadalajara, Tonala, Jalisco, Mexico; ^5^Immunology Research Laboratory, Programa Internacional de Medicina, Universidad Autonoma de Guadalajara, Guadalajara, Jalisco, Mexico

**Keywords:** optic neuropathy, optic neuritis, polyarteritis nodosa, vasculitis, ophthalmic emergency, ophthalmic inflammation

## Abstract

**Purpose:**

To describe a case of optic neuropathy as a primary manifestation of polyarteritis nodosa (PAN) and discuss diagnostic challenges.

**Methods:**

Case report.

**Results:**

A 41-year-old Hispanic man presented with a 2-day history of reduced visual acuity in his left eye. Physical examination revealed a complete visual field loss in the affected eye. Best-corrected visual acuity (BCVA) in the left eye was hand motion, and fundus examination revealed a hyperemic optic disk with blurred margins, swelling, retinal folds, dilated veins, and normal size arteries. BCVA in the right eye was 20/20; no anomalies were seen during examination of the fundus. The patient was started on oral corticosteroids and once the diagnosis of PAN was made, cyclophosphamide was added to the treatment regimen. Six months later, the patient recovered his BCVA to 20/20 in his left eye.

**Conclusion:**

Rarely does optic neuropathy present as a primary manifestation of PAN; nevertheless, it represents an ophthalmologic emergency that requires expeditious anti-inflammatory and immunosuppressive treatment to decrease the probability of permanent visual damage. Unfortunately, diagnosing PAN is challenging as it necessitates a high index of suspicion. In young male patients who present for the first time with diminished visual acuity, ophthalmologists become cornerstones in the suspicion of this diagnosis and should be responsible for continuing the study until a diagnosis is reached to ensure rapid commencement of immunosuppressive treatment.

## Introduction

Polyarteritis nodosa (PAN) presents as a necrotizing vasculitis that affects medium-sized arteries. Histologic examination of an acute lesion typically shows transmural inflammation with a mixed inflammatory infiltrate that is frequently accompanied by fibrinoid necrosis. Small-caliber vessels, such as glomerular and pulmonary capillaries, are not affected (1). PAN has an annual incidence that ranges from 0.9 at Lugo, Spain to 30.7 cases per million adults at Paris, France (2, 3). It affects primarily men around their fourth and sixth decades (4). Pathogenesis of PAN is not completely understood. It may be idiopathic or may be triggered by specific agents such as Hepatitis B virus (HBV) or other viruses. A recent decrease in HBV incidence has been associated with a decrease of new cases of PAN (5), which suggests that the etiology of PAN may be linked to HBV infection. PAN can present as a systemic vasculitis with symptoms including fever, weight loss, myalgias, and arthralgias. Alternatively, the vascular lesions can be restricted to specific organs. The organs most frequently affected are the peripheral nervous system and skin. PAN is also associated with heterogeneous ocular manifestations, such as cotton wool spots, the most prevalent, and optic neuritis (ON), which is less frequent (6).

Polyarteritis nodosa represents a real diagnostic challenge for clinicians, since it is a diagnosis of exclusion. Furthermore, it is important that the diagnosis be made as soon as possible, as expeditious and appropriate treatment is associated with better patient outcome. When the initial manifestation is confined to the eye, the ophthalmologists become a cornerstone in the establishment of the diagnosis. Thus, it is extremely important that ophthalmologists become aware of ocular manifestations in PAN. Here we describe a case of a male patient who developed acute bilateral diminished vision secondary to PAN. More importantly, this patient was diagnosed and treated promptly, showing a significant recovery on visual acuity. Our purpose is to inform ophthalmologists and health-care-associated professionals about the urgency in establishing a diagnosis of PAN, to recognize the diagnostic challenges involved in PAN, and to provide evidence that prompt treatment determines the prognosis on the patient’s visual acuity.

## Case Report

**Figure 1:**
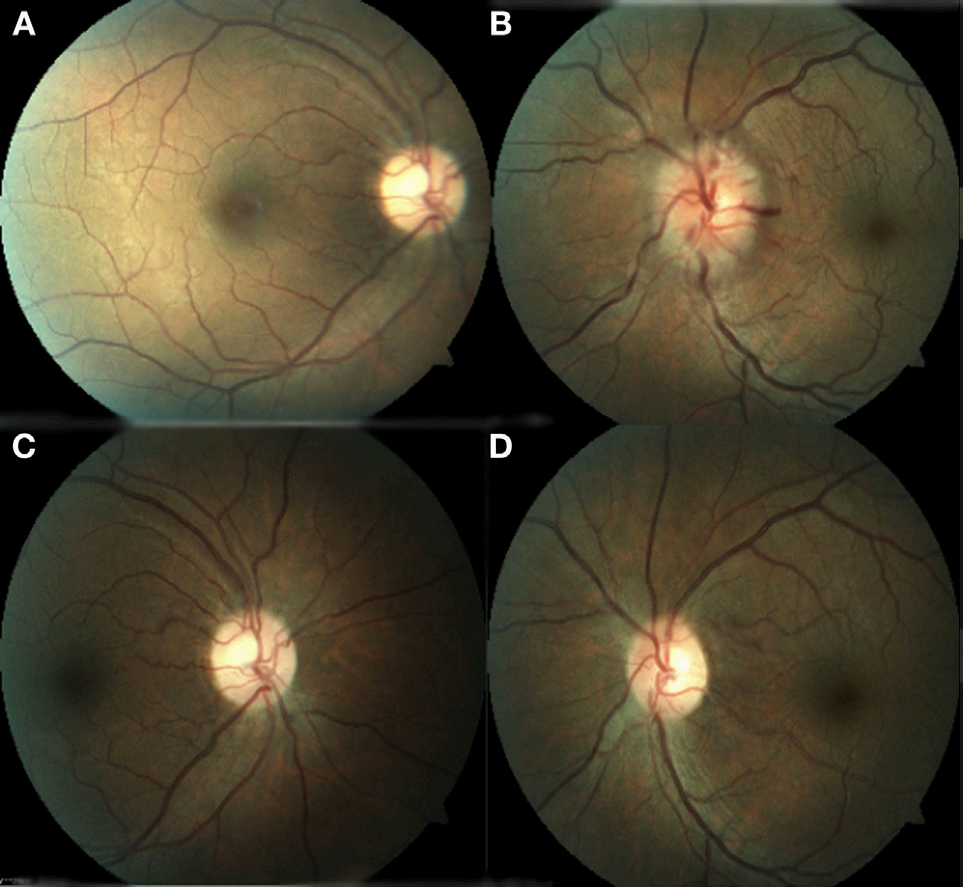
**(A)** Right eye with normal appearance of the optic nerve and macula. **(B)** Left eye with optic nerve hyperemia, swollen, retinal folds, dilated veins, and arteries. **(C)** Right eye with normal appearance of the optic nerve and macula. **(D)** Left eye with normal aspect of the optic disk with retinal and macular folds.

**Figure 2:**
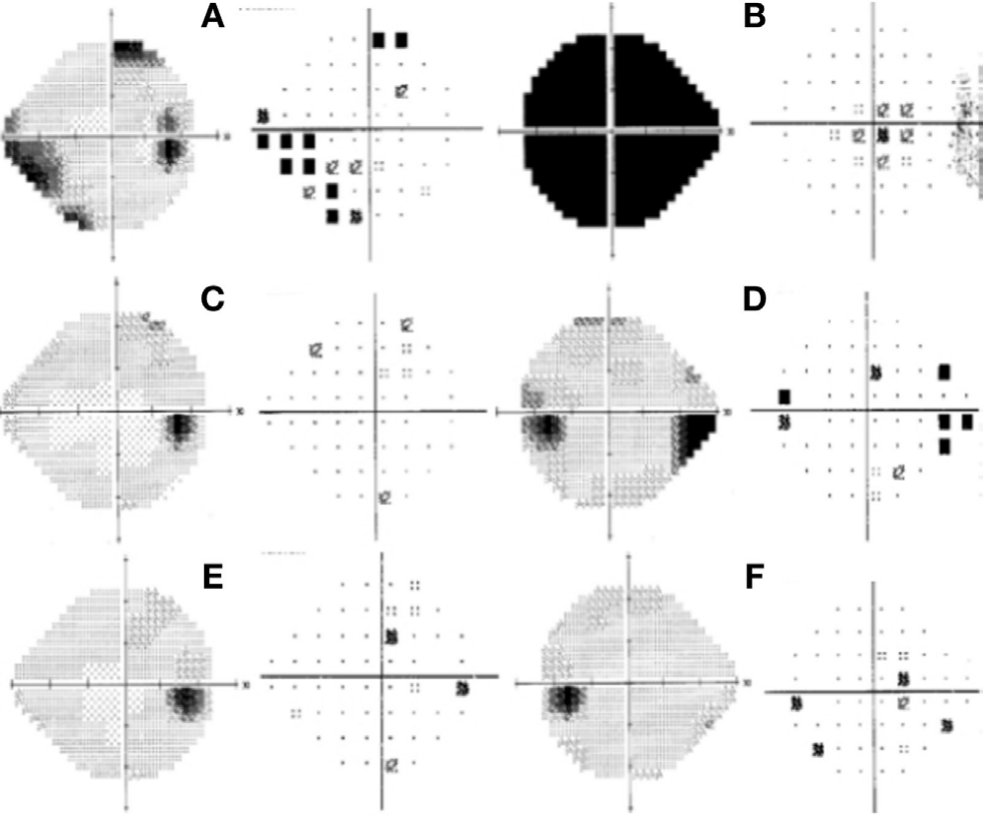
Humphrey visual field test at initial presentation of the right eye; left gray scale right pattern deviation with an inferior-nasal focal depression correlated to a marginal superior-temporal nonspecific scotoma **(A)**. Left eye showed total depression **(B)**. One month after initial presentation: right eye within normal limits **(C)**; left eye with marked superior nasal step with enlargement of blind spot **(D)**. Six months after initial presentation: right eye within normal limits **(E)**. Left eye with some focal point depressed in pattern deviation **(F)**.

A 41-year old Hispanic man presented to the ophthalmology consult with a 2-day history of reduced vision on his left eye, without any known acute inciting factors. His past medical history was significant for a left radical orchiectomy performed 1 year ago secondary to a suspicion of malignancy. Pathological examination of the resected testicle demonstrated fibrinoid necrosis and vascular congestion. During the consult, the patient referred right testicular pain, cutaneous nodules in lower extremities, generalized weakness, and a 2-month weight loss of 8 kg in the previous 4–6 months. He had no history of acute or chronic diseases, and his social history was unremarkable in the context of the present clinical case. On physical examination, blood pressure was 130/98 mmHg. Visual examination revealed best-corrected visual acuity (BCVA) of 20/20 in the right eye and only hand motions associated with relative afferent pupillary defect (RAPD) in the left eye. Bilateral intraocular pressure and anterior segment findings revealed no abnormalities. The right eye had no abnormalities during fundus examination; however, the left optic disk had prominent hyperemia, blurred margins, swelling with retinal folds, dilated veins, and normal sized arteries (Figures 1A,B). Automated perimetry revealed the presence of a focalized inferior-nasal sector depression associated with a marginal superior temporal sector scotoma in the right eye and an extensive visual field defect in the left eye (Figures 2A,B). Unfortunately, fluorescein angiography (FA) was contraindicated, as the patient had a hypersensitivity reaction to fluorescein. A complete blood count, metabolic panel, and urinalysis were performed and all showed no alterations. Venereal disease research laboratory (VDRL) test, purified protein derivative (PPD), anti-HBsAG, and anti-HBcAG were negative. Acute reactants included a normal C-reactive protein of 4 mg/L and an elevated ESR (erythrocyte sedimentation rate) of 28 mm/hr.

The possibility of an inflammatory etiology was then considered and autoantibodies were ordered, including antinuclear antibodies (ANAs), anti-neutrophil cytoplasmic antibodies (c-ANCA), and perinuclear anti-neutrophil cytoplasmic antibodies (p-ANCA). All autoantibody testing was negative. Then, a magnetic resonance image (MRI) with contrast was performed, which revealed thickening of the left optic nerve without evidence of a demyelinating disease.

After integrating information from the patient’s background, clinical manifestations, laboratory results, and ophthalmologic examination, we considered the diagnosis of optic neuropathy and started administration of methylprednisolone 1 gm IV per day for the next 3 days, after which the patient continued with prednisone 75 mg VO for 2 months (tapering doses).

**Figure 3:**
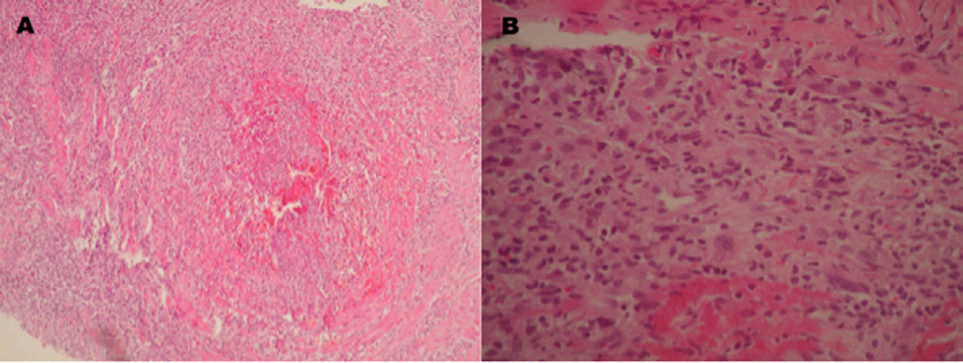
Pathologic examination of a biopsy taken from the left gastrocnemius muscle which shows **(A)** a medium caliber artery with total occlusion due to thrombosis inducing fibrinoid necrosis. **(B)** Inflammatory infiltrate with predominance of polymorphonuclear cells, a few giant multinucleated cells, and fibrinoid deposits.

Seven days after the acute loss in visual acuity, the patient developed a painful nodule on the left gastrocnemius muscle. An excisional biopsy was performed within the next 14 h, which revealed the presence of an occluded medium caliber artery secondary to thrombosis caused by fibrinoid necrosis, an important inflammatory reaction with polymorphonuclear recruitment and no granulomatous lesions (Figure 3).

**Figure 4:**
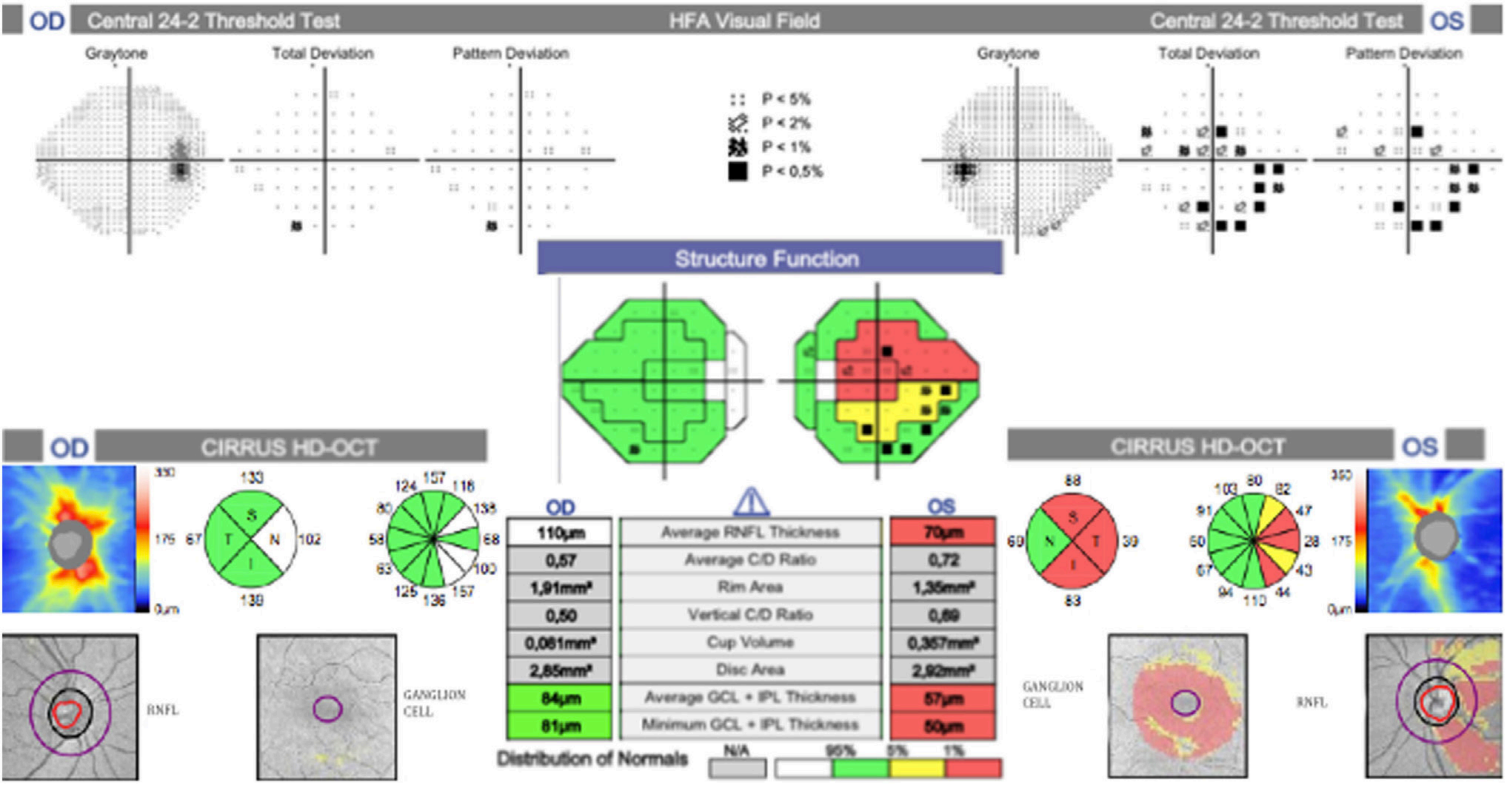
Correlation between the ganglion cell layer (GCL), retinal nerve fiber layer (RNFL), and visual field. Marked thinning of the superior and inferior temporal sectors on RNFL and GCL, which correlates with an inferior and superior scotoma on the visual field.

The patient was then sent to the rheumatology department, where the diagnosis of PAN was established based on the integration of clinical manifestations, laboratory test results, and the exclusion of other pathologies. The prednisone regimen was continued and monthly pulses of intravenous cyclophosphamide (0.6 g/m^2^) were added for the next 6 months. After 1 month of treatment, the patient returned to the ophthalmology consult with clinical improvement, as evident by a BCVA of 20/20 in the right eye and 20/60 in the left eye. Direct ophthalmoscopy of the right fundus revealed no abnormalities, while the left eye had retinal folds involving the macula; however, the optic disk appeared normal. (Figures 1C,D). The patient also demonstrated improvement during visual field examination, with normal appearance in the right eye and marked superior nasal step and enlargement of the blind spot in the left eye (Figures 2C,D). After 6 months of treatment with prednisone and cyclophosphamide, the patient improved his BCVA to 20/20 in the affected eye, with substantial improvement in his visual fields, as shown in Figures 2E,F. Furthermore, the patient had no extraocular manifestations. One year after the first ophthalmologic assessment, a study to compare the areas of macular ganglion cell and retinal nerve fiber layer (RNFL) was performed, which demonstrated a residual thinning of the macular ganglion cells and the nerve fiber layer of the retina, which correlated with the defect of the visual field (pattern deviation) (Figure 4).

## Discussion

### Diagnosis of PAN

Here we present a clinical case of a patient with a systemic affection that manifested clinically as weight loss and fatigue. Systemic affection combined with a history of orchiectomy with fibrinoid necrosis, right testicular pain, cutaneous nodules, slightly elevated ESR, and ANCA negative results directed our thinking to a systemic vasculitis. ANCA antibodies are useful in distinguishing PAN from other systemic vasculitis (microscopic polyangiitis, granulomatosis with polyangiitis, or eosinophilic granulomatosis with polyangiitis), because a negative result is highly suggestive of PAN (7, 8). This finding was important in our clinical case; however, pathological examination and angiographic changes have also a major role in making the diagnosis (8). PAN is a diagnosis of exclusion in ANCA negative patients who requires a careful and histopathologic examination of tissues; therefore, it represents a true diagnostic challenge for clinicians. The American College of Rheumatology (ACR) in 1990 (9) and Chapel Hill Consensus Conference (CHCC) in 1994 (10) proposed classification criteria for PAN and vasculitis, respectively, based on the recognition that histologic data would not be available for all patients. However, these criteria were designed for standardization of research studies and when used for diagnostic purposes, their ability to distinguish vasculitis from other disease is rather poor as they fail to include ANCA (11), or CT-SCAN and MRI, both of which have been proven to be useful in diagnosing vasculitis (12). Nevertheless, the use of ACR 1990 criteria for vasculitis classification was shown to be useful in differentiating between types of vasculitis, with a specificity of 87.8% and sensitivity of 40–6% for PAN (12). Our patient fulfilled 5 out of 10 of these criteria (Table 1).

**Table 1:** American College of Rheumatology (ACR) classification criteria for polyarteritis nodosa (9).

Classification criteria	Clinical case fulfillment
(1) Weight loss > 4 kg	+
(2) Livedo reticularis	−
(3) Testicular pain or tenderness	+
(4) Myalgia’s, weakness, or leg tenderness	+
(5) Mononeuropathy or polyneuropathy	−
(6) Diastolic blood pressure > 90 mmHg	+
(7) Elevated blood urea, nitrogen, or creatinine	−
(8) Hepatitis B virus	−
(9) Arteriographic abnormality	NA
(10) Biopsy of small or medium-sized artery containing polymorphonuclear cells	+

*NA, not available*.

After identifying the diagnostic difficulties inherent in these criteria, a consensus algorithm was developed by Watts and colleagues (8), which combines ACR and CHCC criteria, ANCA testing, and other markers of vascular inflammation. Unfortunately, PAN is situated at the bottom of this algorithm, in which it can only be established after ruling out other diagnoses. Despite combined efforts, criteria for the classification of systemic vasculitis, especially for PAN, remain unsatisfactory, reinforcing the need of international collaborative efforts to update diagnostic protocol.

### Differential Diagnosis of Ocular Manifestations in PAN

Akova et al. accentuated that ocular inflammation can be one of the earliest manifestations in PAN, with a prevalence of 10–20% (13). Other ocular affections include branch and central retinal artery occlusion, ischemic retinopathy, transient monocular visual loss, proptosis, bitemporal and homonymous visual field defect, anterior or posterior ischemic optic neuropathies (ION), and ON (13–15).

Vasculitis within the optic nerve vascular supply (mainly choroidal vessels and posterior ciliary arteries) can cause papillary edema and papillitis, which, in turn, can progress to ION and/or optic nerve atrophy (14–16).

Our patient presented with an acute visual loss and a bilateral optic neuropathy with a severely swollen disk in the left eye. Importantly, there was a rapid recovery secondary to anti-inflammatory and immunosuppressive therapy, which demonstrated empirically improved disk appearance and an augmented visual field. Based on these findings, we hypothesize that our patient developed an atypical bilateral ON secondary to PAN. Unfortunately, our diagnosis could not be confirmed with histopathologic examination of the optic nerve or angiographic tests. Regardless, ION was discarded due to the fact that although patients can display similar manifestations (17), visual loss and prognosis are often worse since the damage to the optic nerve is irreversible, causing progressive visual loss characterized by a pale and swollen optic disk (18, 19). Additionally, disk edema tends to be sectoral in the affected eye and usually there is a small cup-to-disk ratio, whereas the less or non-affected eye will present with a normal or enlarged cup (20), which was absent in our case. After FA, choroidal hypoperfusion and delayed choroidal filling can be observed (21). Unfortunately, in our case, FA was contraindicated; however, an optical coherence tomography (OCT) performed 1 year after the optic neuropathy episode demonstrated thinning of nerve fiber layers and of ganglion cells. These pathological changes have previously been reported to occur after ON (22). OCT is a novel study that allows a quantitative assessment of the thickening in ganglion cells layers and RNFLs. With this information, it is possible to correlate the degree of slimming within these layers to abnormalities in the visual fields. Although its use is still experimental and we are not certain of the pathologic processes involved in the slimming, results from this test provide valuable information in cases where is not possible to perform FA, as it was in our case. Additionally, the use of such novel diagnostic tools allows us to develop our understanding of the structural and functional changes that occur during the course of ON.

Additionally, ON usually presents with periocular pain, yet our patient referred no pain at all. In some cases, spontaneous recovery may start within the first 3 weeks after the acute onset (23), although the mechanism is not fully described. ON is a demyelinating inflammation of the optic nerve usually accompanied by unilateral or bilateral visual field defects. Even with resolution, there is a possibility of recurrence (24). The etiology of ON is mainly idiopathic, although it has been associated with demyelinating conditions (multiple sclerosis), autoimmune diseases, infectious diseases, and even vaccination (25). After a long-term follow-up study, Kurne et al. described 70 cases of ON with several etiologies. In total, 47 patients had unilateral ON and 23 presented with bilateral affection. Among the later, vasculitides were the most frequent etiology (57%), including systemic lupus erythematosus, Sjogren’s syndrome, and PAN (24). To our knowledge, this is the only previous study that reported ON as a manifestation of PAN (24). We are not certain if the low number of bibliographic references reflects a low prevalence of ON secondary to PAN or if it is due to difficulties in establishing the diagnosis before it gets complicated with necrosis, since ION has been slightly more frequently reported in PAN compared to ON (26–28).

### Immunopathogenesis of Ocular Manifestations in PAN

It was postulated that in PAN exposure to viral antigens triggers the complement cascade, resulting in the liberation of chemotactic factors for neutrophils and lymphocytes within the arterial media, which, in turn, can cause fibrosis, thrombosis, or aneurysmal degeneration (29). The recently described antibodies against endothelial cells (AECAs) may also play a role in the development of PAN, since they can trigger the activation of the complement system by the classical pathway (30), induce antibody-dependent cell cytotoxicity, activate endothelial cells to upregulate the expression of adhesion molecules, and induce the production of cytokines and chemokines. However, neither their exact role in PAN has been fully elucidated nor the mechanisms by which antibodies or immune complexes lead to inflammation in small and medium-sized arteries. Irrespective of the initial trigger for inflammatory cell recruitment, in general, vasculitides are characterized by inflammatory cell infiltration in the arterial media with subsequent disruption of the internal elastic lamina. Obstruction of arterial lumen may result from either thickening of the intima, edema, or thrombosis (31). Within the eye, it is important to acknowledge that the vasculitic process is not limited to the retinal circulation: it can present as conjunctivitis episcleritis, scleritis, uveitis, or, as in this case, neuritis. During the autopsy of a patient with optic nerve affection secondary to PAN, vasculitis was identified in short posterior ciliary arteries and other orbital arteries (32). Unfortunately, the case was published in 1974, when other diagnostic tools were not available.

### Treatment of Ocular Manifestations in PAN

In general, treatment of vasculitides relies on the use of potent anti-inflammatory drugs. Corticosteroids are considered the first line of treatment; they can be given orally or intravenously (33). Cyclophosphamide is usually added to the treatment regimen when there is critical organ involvement (1). Our patient’s rapid recovery, demonstrated through visual field and fundus eye examination, can be attributed to immediate administration of corticosteroids and cyclophosphamide, which was possible due to our prompt diagnosis of ON secondary to PAN. We acknowledge that the prompt diagnosis of PAN may not be the norm, since the establishment of the correct diagnosis is inherently challenging.

In severe cases of PAN, mortality remains high despite the appropriate treatment (4–22%) (34), since treatment-related toxicities are common. Alternatives for treatment include mycophenolate mofetil (MMF), which has been used in other systemic vasculitis as an alternative to cyclophosphamide, especially in the pediatric population (35). The use of biologic agents, such as anti-TNFα, is not formally indicated in the treatment of PAN, but could be options for patients with severe side effects (36).

## Conclusion

Here, we present a case with severe anatomic and functional ocular affection secondary to PAN, which was diagnosed only after thorough consideration of signs, symptoms and laboratory test results, and with the active participation of rheumatologists. Our patient had an exceptional recovery due to swift commencement of anti-inflammatory and immunosuppressive treatment. It must be stressed, however, that the diagnosis of PAN with isolated ophthalmologic affection represents a challenge, since it requires a high degree of suspicion, specialized tests, and histopathologic examination to finally confirm the diagnosis. Unfortunately, a prompt diagnosis is of extreme importance, since the sooner an appropriate treatment is initiated, the better the prognosis and the less probability of permanent visual damage. It is important to highlight the need for new research studies that aim to identify biomarkers that might be involved in PAN development and that can be used to improve our diagnostic abilities. ON secondary to PAN should be suspected in male patients around their fourth decade who present with sudden visual loss, nonspecific systemic affection, such as weight loss and fatigue, and who lack inciting factors involved in acute loss of vision. In these cases, ophthalmologists play a crucial role in establishing the correct diagnosis and therefore it is imperative that they be made aware of ophthalmic pathologies secondary to PAN, the diagnostic challenge associated with PAN, and the need for rapid commencement of immunosuppressive treatment.

## Ethics Statement

Written Informed Consent to publish the report was obtained from the patient.

## Author Contributions

All authors attest that they meet the current ICMJE criteria for Authorship.

## Conflict of Interest Statement

The authors report no conflicts of interest. The authors alone are responsible for the content and writing of the paper. The reviewer, AR, declares a shared affiliation with the editor.
